# Otoplasty for prominent ear: A systematic review of surgical techniques

**DOI:** 10.1016/j.jpra.2026.02.013

**Published:** 2026-02-23

**Authors:** Hamish Thomson, Jack Gosden, Anirban Mandal

**Affiliations:** a School of Medicine, University of Liverpool, Liverpool, UK; b The Mersey Regional Burns and Plastic Surgery Unit, Mersey and West Lancashire Teaching Hospitals NHS Trust, Knowsley, UK

**Keywords:** Otoplasty, Prominent ear, Superior, Technique, Comparison

## Abstract

Prominent ear is a common auricular malformation that can have long-term psychosocial consequences on affected individuals. Otoplasty remains the standard surgical corrective treatment; however, the optimal technique has long been debated, with >200 methods described in the literature. This systematic review, conducted in accordance with PRISMA guidelines, aimed to evaluate the available evidence on the most effective otoplasty approach. The outcomes assessed across different techniques (exclusive suture, exclusive cartilage-scoring, incisionless, suture + flap, suture + cartilage-scoring, and cartilage-scoring + flap hybrids) included: a) number of patients suffering a complication, b) recurrence, c) reoperation, d) infection, e) keloid or hypertrophic scar formation, and f) haematoma. A search of PubMed and MEDLINE identified 412 papers. Following PICOT framework-guided screening, application of inclusion and exclusion criteria, and quality assessment, 22 studies were included: 19 retrospective and three prospective cohort studies. Current medical literature suggests that suture + cartilage-scoring and cartilage-scoring + flap hybrid methods, may be associated with lower rates of recurrence, and reoperation when compared to single-technique or suture + flap approaches. However, high-quality, long-term randomised control trials are required to determine the superior technique for otoplasty.

## Introduction

Protruding ear is one of the most common auricular deformities affecting the Caucasian population, with approximately 5% believing that their ears excessively protrude.^1^ It features inadequate folding of the antihelix and/or increased conchal depth, leading to excessive projection of the auricle, typically >2 cm from the mastoid, or with an auriculocephalic angle >25°. The aesthetic and psychosocial consequences of protruding ear can be detrimental to the individual and is known to lower self-esteem, increase social anxiety and in the case of children, increase their susceptibility to bullying.^1^

Otoplasty is the mainstay of treatment to correct prominent ear. With over 200 otoplasty techniques being described in the current literature, it can be challenging for surgeons to select the optimal method for their patients.^2^ An otoplasty typically starts with a postauricular incision being made within the auriculomastoid sulcus to access the auricular cartilage. From this point, many techniques exist to recreate the antihelical fold and/or set back the conchal bowl.^3^

• Cartilage-sparing (suture-based) techniques such as Mustardé^4^ utilise mattress sutures to create the anti-helical fold. To achieve a balanced correction, this method is often performed in combination with another suture-based technique, Furnas,^5^ which employs concha-mastoid mattress sutures to set back the auricle by narrowing the mastoid helical angle. This approach is best utilised when the auricular cartilage is immature and pliable such as that found in children, however in older patients with firmer cartilage, recurrence is more common due to suture cut-through and/or cartilage memory.^6^
• Cartilage-scoring techniques, pioneered by Stensröm,^7^ Crikelair^8^ and Chongchet^9^ exploit the bio-mechanical mechanism that cartilage over time, naturally bends away from the side of scoring. Scoring, or thinning the cartilage allows controlled formation of an anti-helical fold which is particularly advantageous in patients with thick or rigid cartilage. Excessive or uneven scoring risks the production of sharp edges and/or chondronecrosis.^6^
• Incisionless techniques first described by Fritsch^10^ revolve around the percutaneous placement of mattress sutures through needle punctures, negating the need for large skin incisions and theoretically reducing the risk of haematoma, necrosis and keloid/hypertrophic scarring. Similar to suture-based techniques, it is optimal to be used in the younger population.^10^
• The suture-postauricular fascial flap hybrid is a method piloted by Horlock et al.^11^ that introduces a postauricular flap to provide a vascularised layer over the sutures, thereby theoretically reducing the risk of extrusion and recurrence, a notorious limitation of using sutures exclusively.^11^
• Suture-cartilage scoring hybrids combine posterior suturing with minimal anterior cartilage-scoring to achieve a superior fold, while reducing the risks of suture extrusion associated with sutures alone, and the sharp edges or weakening linked to cartilage-only method.^12^
• Cartilage scoring-flap hybrids as described by Scuderi et al.^13^ combine posterior chondro-muscular flap advancement with anterior cartilage scoring. The flap narrows the auriculocephalic angle, and adjusts the auricular slope, while cartilage scoring or cutting recreates the antihelical fold. This dual approach enhances stability and contour while reducing risks of recurrence, or unnatural rigidity that can be seen with single-technique methods.^13^

Several complications are associated with otoplasty, both in the short term such as bleeding, infection, haematoma and necrosis, and the long term, including recurrence, reoperation, suture extrusion, asymmetry and poor aesthetic results. These complications increase the risk of prolongating the negative psychological impact that prominent ear can have on patients. There is no up-to-date comparison between techniques, necessitating the need for a review.^2^

This systematic review was conducted in accordance with PRISMA guidelines,^14^ and followed the population, intervention, comparison, outcome and time (PICOT) framework.^15^ The review compares traditional techniques, exclusive suture or cartilage-scoring methods, with more recent approaches, including incisionless, and hybrid techniques (suture + flap, suture + cartilage-scoring, and cartilage-scoring + flap). The study population comprised patients undergoing otoplasty for the correction of prominent ear. The primary intervention of interest was the use of suture-only or cartilage-scoring techniques, with comparators including incisionless and hybrid approaches. Outcomes assessed included a) number of patients suffering a complication, b) recurrence, c) reoperation, d) infection, e) keloid or hypertrophic scar formation, and f) haematoma. These outcomes were evaluated in both the short term (<1 year) and long term (>1 year). This review aims to provide a reference for plastic surgeons in selecting the most appropriate otoplasty technique for patients with prominent ear. We hypothesised that suture-based techniques combined with a postauricular fascial flap represent the most effective method for achieving durable correction.

## Methodology

A systematic search of the medical databases PubMed and MEDLINE^16^^,^^17^ was conducted without the assistance of a medical librarian on 1 September 2025, using search strategies specific to each database, adhering to PRISMA guidelines^14^ and following the PICOT framework.^15^ PubMed and MEDLINE were selected for their extensive coverage of high-quality studies published in the fields of medicine and surgery. The MEDLINE search was performed using the OVID search engine.^18^ Only papers published between 1 January 2001, and 1 September 2025 were selected to ensure the review aligns with modern otoplasty techniques. The precise search terms and Boolean operators used for each database are detailed in the appendix, within Table 1, Table 2. All types of study were included in the search strategy. Following the search, all identified studies were uploaded to the latest version of the AI systematic review software Rayyan^19^ and 2 authors (H.T. and A.M.) evaluated the abstracts against the PICOT framework-guided^15^ inclusion criteria. Additionally, the bibliography of each article was screened to identify further relevant studies.

**Table 1:** PubMed search strategy, date – 01/09/2025.

Number	Term	Results
#1	(otoplasty [tiab])	674
#2	(prominent ears [tiab]) OR (prominent ear [tiab])	622
#3	#1 OR #2	1042
#4	(management [tiab])	1,737,902
#5	(comparison [tiab])	1,325,810
#6	(outcome [tiab])	1,440,532
#7	#4 OR #5 OR #6	4,195,520
#8	#3 AND #7	191
#9	#3 AND #7 Filter: from 1/1/2001 to 1/9/2025	165

**Table 2:** MEDLINE search strategy date – 01/09/2025.

Number	Term	Results
#1	otoplasty.mp	674
#2	prominent ears.mp. OR prominent ear.mp	621
#3	#1 OR #2	1041
#4	management.mp	1,898,364
#5	comparison.mp	1,349,856
#6	outcome.mp	2,581,434
#7	#4 OR #5 OR #6	5,327,788
#8	#3 AND #7	287
#9	Limit #8 to yr = “2001–2025”	247

The selection process was guided by the PICOT framework to ensure optimal comparison between otoplasty outcomes^15^:

• Population (P) – Patients undergoing otoplasty to correct prominent ear.
• Intervention (I) – Otoplasty exclusively using cartilage-sparing (suture-based) or cartilage-scoring techniques.
• Comparison (C) – Otoplasty using incisionless, or suture-flap, suture-cartilage scoring or cartilage scoring-flap hybrids.
• Outcome (O) – a) Number of patients suffering a complication, b) Recurrence, c) Reoperation, d) Infection, e) Keloid or hypertrophic scar, f) Haematoma.
• Time (T) – Both short-term (≤1 year) and long term (≥1 year).

### Inclusion criteria

The paper must:

• Include primary data.
• Include human participants.
• Be comprised of ≥50 patients.
• Have an identifiable study design.
• Involve one of, or a combination of suture-based, cartilage-scoring, incisionless or hybrid technique to perform otoplasty to correct prominent ear.
• Be available in English.
• Be fully accessible to the authors.
• Be a full study.
• Meet high ethical standards.

### Exclusion criteria

The paper must not:

• Be a systematic or literature review.
• Be published prior to 1 January 2000.
• Have ≤50 patients*.

**Figure 1:**
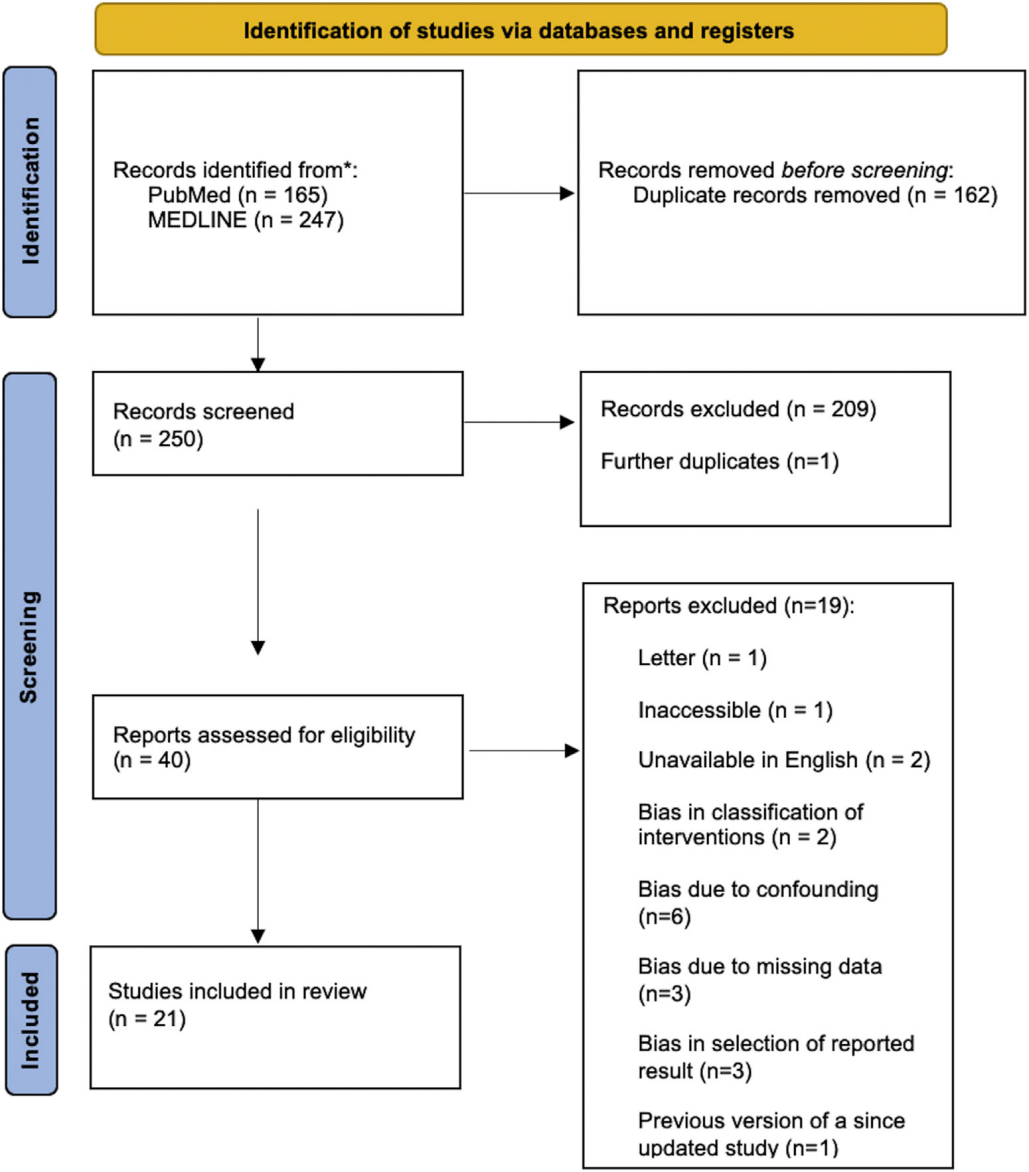
PRISMA flowchart.

Summarised in Figure 1,^20^ the original search identified 412 studies (PubMed = 165; MEDLINE = 247). Following the removal of 162 duplicates, 250 papers were independently assessed by authors H.T. and A.M. against the inclusion and exclusion criteria, and PICOT framework. A total of 209 papers were excluded, and one additional duplicate was identified, leaving 40 papers to be put through quality assessment. The quality and risk of bias in each study were evaluated using the Risk of Bias in Non-Randomised Studies of Interventions, Version 2 (ROBINS-I V2) tool.^21^ Studies with a moderate risk of bias were accepted due to the limited availability of randomised controlled trials (RCTs). A total of 19 papers were excluded during the quality assessment process, leaving 21 papers included in the final review.

The characteristics of each study are detailed in Table 3. Patients that underwent each method of otoplasty were identified and compiled into a sub-analysis. Patient characteristics, confounding factors, and each of the primary outcomes; a) Number of patients suffering a complication, b) recurrence, c) reoperation, d) infection, e) keloid or hypertrophic scar and f) haematoma were stratified for each method of otoplasty and displayed in Table 4. Studies reporting mean values were grouped together and a cumulative mean was calculated. Similarly, studies reporting median values were grouped and a cumulative median was calculated.

**Table 3:** Study characteristics.

Study	Location	Single or multi centre	Study type	Sample	Ears	Median (Range)/Mean (±SD) Age, years	Gender, *n* (%)	Method(s) of otoplasty, *n* (%)	Follow-Up, median (Range) or mean (± SD), months
(Grella et al., 2025)	Italy	Single	Retrospective	50	100	20 ± 11.5	M – 24 (48) F – 26 (52)	CS – 50 (100)	33.9 ± 16.1
(Gualdi et al., 2025)	Italy	Single	Retrospective	146	292	23.2 ± 5.3	M – 81 (55.5) F – 65 (44.5)	CS – 146 (100)	22
(Gilron et al., 2024)	Israel	Single	Retrospective	147	288	16.6 ± 9.9	M – 56 (38.1) F – 91 (61.9)	SB – 147 (100)	24.3 ± 11.2
(Kadhum et al., 2024)	UK	Single	Retrospective	203	372	13 ± 4	M – 103 (51) F – 100 (49)	SB – 194 (96) CS – 9 (4)	12
(Uyar et al., 2023)	Turkey	Single	Prospective	52	104	14.3 ± 5.8	M – 20 (38.5) F – 32 (61.5)	SB – 52 (100)	12
(Binet et al., 2020)	Switzerland	Single	Retrospective	705	1380	10.4 ± 2.9	M – 266 (37.8) F – 439 (62.2)	*S* + CS – 705 (100)	132 ± 72
(García-Purriños et al., 2019)	Spain	Single	Retrospective	172	343	16.2 ± 14	M – 61 (35.3) F – 111 (64.5)	SB – 172 (100)	18
(Brian et al., 2019)	NZ	Single	Retrospective	64	117	9.5 ± 4.2	M – 29 (45.3) F – 35 (54.7)	CS – 64 (100)	32.7 ± 17.1
(Ersen, 2019)	Turkey	Single	Retrospective	162	322	28.3 (18–40)	M – 141 (87) F – 21 (13)	*S* + PF – 162 (100)	22.5 ± 7.5
(Haytoglu et al., 2015)	Turkey	Single	Prospective	60	112	8.3 ± 2.8	M – 34 (56.6) F – 26 (43.3)	INCL – 60 (100)	NR
(Toplu et al., 2014)	Turkey	Single	Prospective	77	132	14.6 ± 6.4	M – 18 (45) F – 22 (55)	SB – 40 (51.9) CS – 37 (48.1)	8
(Park and Jeong, 2012)	SK	Single	Retrospective	66	90	23.7 ± 10.1	M – 27 (40.9) F – 39 (59.1)	*S* + CS – 66 (100)	62.4 ± 12
(Maricevich, 2011)	Brazil	Single	Retrospective	111	218	28.2 ± 15	M – 31 (28) F – 80 (72)	CS – 111 (100)	NR
(Schaverien, 2010)	UK	Single	Retrospective	60	112	8.5 ± 2.5	M – 34 (56.6) F – 26 (43.3)	*S* + PF – 60 (100)	46.8 ± 18.9
(Olivier et al., 2009)	Canada	Single	Retrospective	104	203	7.1 ± 3.5	M – 64 (62) F – 40 (38)	SB – 104 (100)	99
(Scharer et al., 2007)	USA	Single	Retrospective	75	144	23.9 ± 15.8	M – 40 (53) F – 35 (47)	*S* + CS – 73 (97.3) N/A – 2 (2.7)	12 ± 21.4
(Salgarello et al., 2007)	Italy	Single	Retrospective	135	266	13 ± 7.5	M – 51 (37.7) F – 84 (62.3)	*S* + CS – 135 (100)	60 ± 33
(Scuderi et al., 2007)	Italy	Single	Retrospective	55	103	14 (6–55)	M – 38 (47.5) F – 42 (52.5)	CS + *F* – 55 (100)	14.3 (12–36)
(Mandal et al., 2006)	UK	Single	Retrospective	203	406	9 (5–16)	M – 127 (62.6) F – 76 (37.4)	SB – 94 (46.3) CS – 68 (33.5) *S* + PF – 41 (20.2)	11 (9–36)
(Bulstrode et al., 2003)	UK	Single	Retrospective	114	214	18.3 ± 15.8	M – 57 (50) F – 57 (50)	CS – 114 (100)	47 ± 26.3
(Yugueros and Friedland, 2001)	USA	Single	Retrospective	100	193	38 ± 15.5	M – 29 (29) F – 71 (71)	*S* + CS −100 (100)	40 ± 51

Key – CS, Cartilage Scoring Technique; CS + F, Cartilage Scoring + Flap Hybrid; F, Female; M, Male; IMP, Implant Technique; INCL, Incisionless Technique; N/A, Unknown; NR, Not Reported; NZ, New Zealand; SB, Suture-Based Technique; SK, South Korea; S + CS, Suture + Cartilage Scoring Hybrid; S + PF, Suture-Postauricular Flap Hybrid; UK, United Kingdom; USA, United States of America.

**Table 4:** Sub analysis of otoplasty techniques.

	Suture-based (*n* = 812)	Cartilage-scoring (*n* = 590)	Incisionless (*n* = 60)	Suture + Flap (*n* = 263)	Suture + Cartilage-scoring (*n* = 1074)	Cartilage-scoring + Flap (*n* = 55)
Age Cumulative median (Range) Cumulative mean (± SD)	11 (9–36) 13.6 ± 7.3	9 (5–16) 18.9 ± 9.7	- 8.3 ± 2.8	18.7 (5–40) 8.5 ± 2.5	- 21.8 ± 13.1	14 (6–55) -
Gender, *n* (%) Male Female	381 (46.9) 431 (53.1)	283 (48) 307 (52)	34 (56.6) 26 (43.3)	201 (76.4) 62 (23.6)	406 (37.8) 668 (62.2)	38 (47.5) 42 (52.5)
Ears, *n*	1566	1141	112	516	2072	103
Follow up, months Cumulative median (Range) Cumulative mean (± SD)	11 (9–36) 28.9 ± 11.2	11 (9–36) 28.3 ± 11.9	- 6	11 (9–36) 34.7 ± 13.2	- 50.5 ± 37.9	14.3 (12–36) -
Confounding factors, *n* (%)						
Smoker	20 (2.5)	8 (1.4)	NR	NR	NR	NR
Patients suffering a complication, *n* (%)	208 (25.6)	121 (20.5)	15 (25)	36 (13.7)	112 (10.4)	2 (3.6)
Recurrence, *n* (%)	45 (5.5)	49 (8.3)	6 (10)	24 (9.1)	36 (3.4)	2 (3.6)
Reoperation, *n* (%)	37 (4.6)	39 (6.6)	6 (10)	21 (7.9)	42 (3.9)	2 (3.6)
Infection, *n* (%)	8 (0.9)	6 (1.02)	0	0	0	0
Keloid or hypertrophic scar, *n* (%)	18 (2.2)	10 (1.7)	0	0	22 (2.1)	0
Haematoma, *n* (%)	9 (1.1)	3 (0.5)	0	1 (0.4)	1 (0.1)	0

Key – NR, Not Reported.

## Results

### Study characteristics

A total of 21 studies were included in this review, comprising 18 retrospective cohort studies,^12^^,^^13^^,^^22^, ^23^, ^24^, ^25^, ^26^, ^27^, ^28^, ^29^, ^30^, ^31^, ^32^, ^33^, ^34^, ^35^, ^36^, ^37^ and three prospective cohort studies.^38^, ^39^, ^40^ The studies were conducted across a diverse range of countries. Sample sizes ranged from 50 to 705^12^^,^^22^ with a median of 104 and a mean of 136.2 participants. Only patients who underwent an otoplasty technique relevant to this review were included in the reported sample sizes. The number of ears analysed ranged from 90 to 1380, with a median of 214 and a mean of 254.5. All studies were single-centre in design. Among studies reporting follow-up duration using median values, the pooled median was 12.65 months (range 9–36), while the pooled mean follow-up duration was 17 ± 8.5 months.

### Method of otoplasty

A total of 812 patients underwent otoplasty using exclusively suture-based techniques, of whom 381 (46.9%) were male. Cartilage-scoring techniques alone were performed in 590 patients, including 283 (48%) males. The incisionless approach was used in 60 patients, with 34 (56.6%) males. A suture + flap hybrid technique was employed in 263 patients, including 201 (76.4%) males. Suture + cartilage-scoring hybrids were used in 1074 patients, with 406 (37.8%) males. Finally, a cartilage-scoring + flap hybrid was performed in 55 patients, 38 (47.5%) of whom were male.

### Number of patients suffering a complication

**Figure 2:**
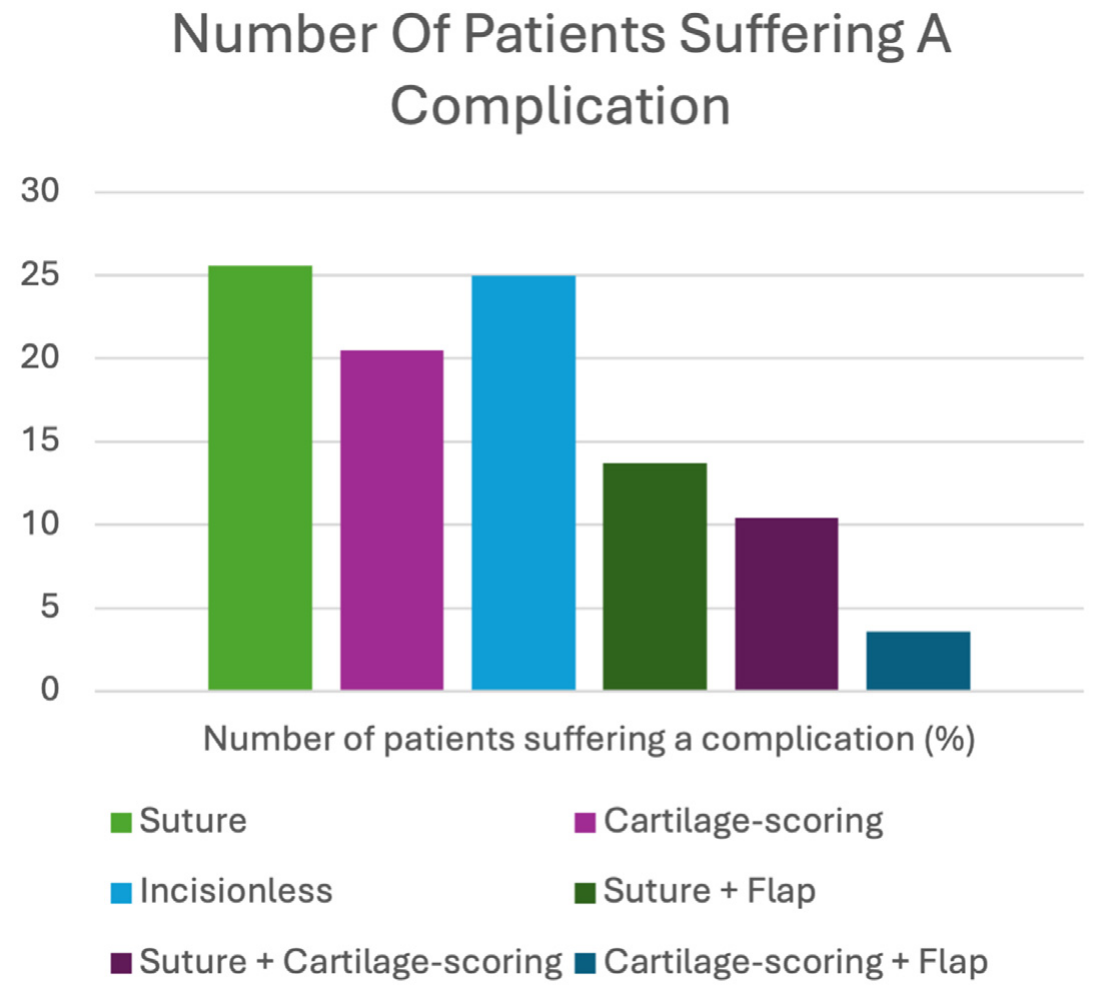
Number of patients suffering a complication.

The highest proportion of patients suffering a complication was associated with the exclusive use of suture-based techniques, with 25.6% of patients affected; incisionless techniques followed with a rate of 25%. Exclusive cartilage-scoring approaches resulted in complications in 20.5% of patients. In contrast, hybrid techniques demonstrated lower numbers of patients suffering a complication: 13.7% for suture + flap hybrids, 10.4% for suture + cartilage-scoring hybrids, and 3.6% for cartilage-scoring + flap hybrids. The total number of patients suffering a complication from each method of otoplasty is summarised in Figure 2.

### Recurrence

**Figure 3:**
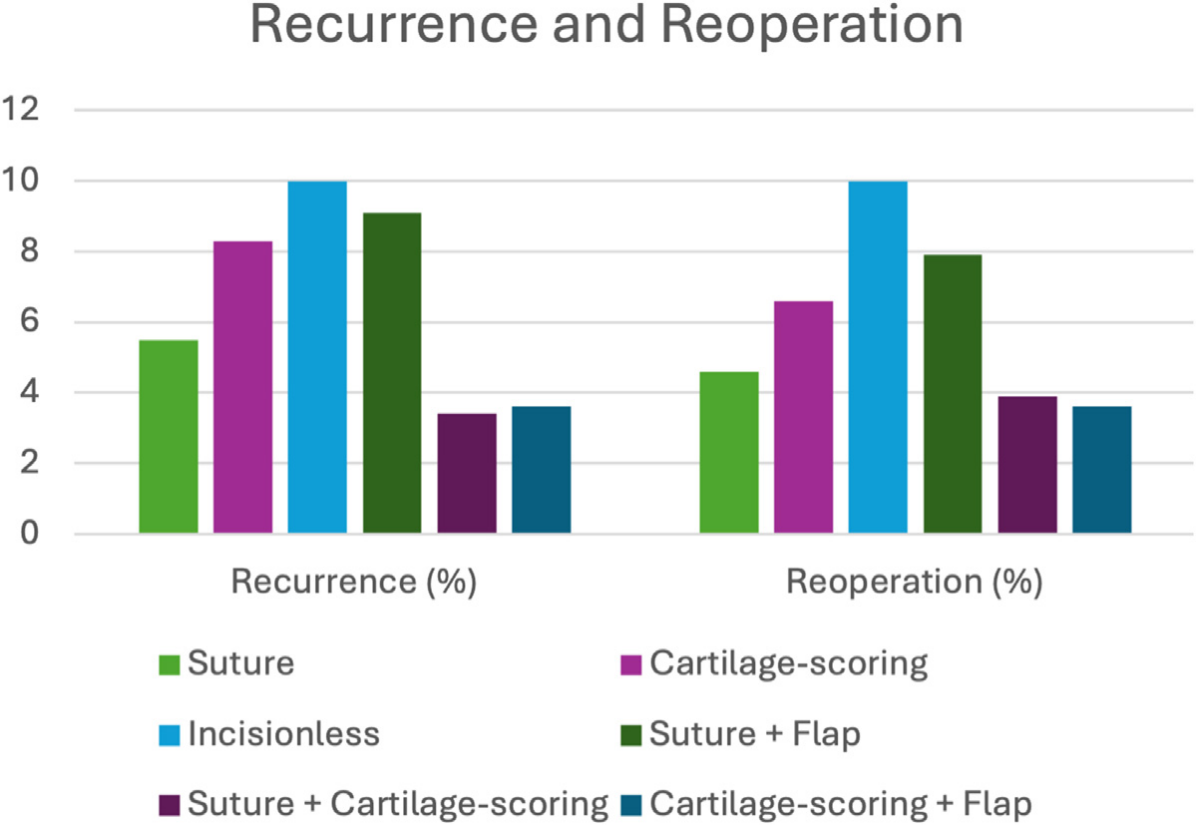
Recurrence and reoperation for each method of otoplasty.

Recurrence was consistently reported across the included studies. Overall, recurrence was ≤ 10% for all otoplasty techniques, with the incisionless method reporting the highest (*n* = 10%). Among patients who underwent isolated suture or cartilage-scoring techniques, recurrence was observed in 5.5% and 8.3% of cases, respectively. The suture + flap hybrid technique was associated with a recurrence rate of 9.1%. The lowest recurrence rates were reported with suture + cartilage-scoring (3.4%) and cartilage-scoring + flap (3.6%) hybrid techniques. The recurrence rates for each method of otoplasty are displayed in Figure 3.

### Reoperation

Reoperation rates, whether due to recurrence, asymmetry, patient dissatisfaction, or other concerns, were consistently reported across the included studies. The highest rate was observed in the incisionless cohort, with 10% of patients requiring a secondary procedure. Exclusive suture and cartilage-scoring techniques required further surgery in 4.6% and 6.6% of cases, respectively; suture + flap hybrids and suture + cartilage-scoring hybrids in 7.9% and 3.9%, respectively. The lowest rate was reported in the cartilage-scoring + flap hybrid group, with 3.6% of patients requiring reoperation. The reoperations rates for each method of otoplasty are shown in Figure 3.

### Infection

Overall, infection rates were low across all methods. No postoperative infections were reported among the hybrid techniques (suture + flap, suture + cartilage-scoring, and cartilage-scoring + flap), or incisionless methods. Exclusive cartilage-scoring and suture-based techniques each reported an infection rate of 1.02% and 0.9% respectively.

### Keloid or hypertrophic scar formation

Similar to infections, keloid or hypertrophic scar formation was relatively uncommon across the review. No cases were reported in the incisionless cohort or among patients undergoing the suture + flap or cartilage-scoring + flap hybrid techniques. Exclusive cartilage-scoring resulted in keloid or hypertrophic scars in 1.7% of patients. Exclusive suture-based techniques and suture + cartilage-scoring hybrids demonstrated comparable rates, at 2.2% and 2.1%, respectively.

### Haematoma

Haematomas were also uncommon. No cases were observed in the cartilage-scoring + flap hybrid group nor those undergoing an incisionless technique. Haematomas occurred in 0.6% of patients treated with exclusive suture techniques and 0.5% with exclusive cartilage-scoring. The suture + flap and suture + cartilage-scoring hybrids reported rates of 0.4% and 0.1%, respectively.

## Discussion

This systematic review aimed to explore the available medical literature on the optimal technique of otoplasty for correction of prominent ear. Several key observations were made in the review.

The literature indicates that suture + cartilage-scoring and cartilage-scoring + flap hybrid techniques represent the most effective methods of otoplasty. These techniques were associated with lower rates of recurrence and reoperation compared to other methods. Notably, the suture + cartilage-scoring approach had the largest reported sample size (*n* = 1074), strengthening the evidence for its efficacy in the management of prominent ear. Although fewer patients undergoing suture–flap hybrid otoplasty experienced complications compared with exclusively suture-based and cartilage-scoring techniques, a higher proportion of these complications being recurrence and/or reoperation was observed. This suggests that the suture-flap hybrid technique may be associated with the greatest risk of recurrence and/or reoperation amongst hybrid methods of otoplasty, which differs from our hypothesis that the suture + flap hybrid would have superior outcomes. The evidence base for incisionless otoplasty remains limited due to small sample size and reported outcomes appearing less favourable compared with other methods. This is emphasised by the incisionless technique demonstrating the highest rates of recurrence and reoperation.

Traditional techniques, such as the exclusive use of sutures and/or cartilage-scoring, demonstrated higher incidences of recurrence and reoperation than suture + cartilage-scoring and cartilage-scoring + flap hybrid techniques, however, demonstrated a lower recurrence and reoperation rate than incisionless and suture + flap hybrid methods. Notably, the exclusive use of sutures was associated with the highest proportion of patients experiencing a complication in the review. This is likely due to the increased incidence of minor complications such as suture extrusion and/or irregularity of the contour fold. This suggests that, while traditional techniques may provide adequate correction of prominent ear, combining them either with each other, or cartilage-scoring with a vascularised flap, appears to reduce the number of patients suffering a complication, and thereby improve overall patient outcomes.

In comparison to a review on the same topic, Sadhra et al.^41^ evaluated the average incidence of complications following otoplasty and identified the exclusive use of sutures as the method associated with the highest rates of recurrence and reoperation.^41^ Unlike the present review, their analysis was limited to suture-only, cartilage-scoring, and suture + cartilage-scoring hybrid techniques. Moreover, Sadhra et al. predates the publication of several studies included in the current review. This emphasises the need for an updated review that incorporates the full range of otoplasty methods currently in practice.

To accurately determine the superior method of otoplasty or confirm suture + cartilage-scoring and/or cartilage-scoring + flap methods as the superior technique, long-term RCTs are required, with comprehensive reporting on patient characteristics, and clinical outcomes per patient. All studies included in this review adhered to the ethical guidelines, provided appropriate follow-up care and, in several cases, reported long-term outcome monitoring, reflecting a commitment to the highest principles of medical research ethics.

## Conclusion

In conclusion, although this review is limited by the absence of randomised controlled trials (RCTs), inconsistent stratification and outcome reporting, and unequal sample sizes within the comparative groups in the sub-analysis, the available evidence suggests that suture + cartilage-scoring and/or cartilage-scoring + flap hybrid approaches to otoplasty may offer superior outcomes. These methods are generally associated with lower rates of recurrence, and reoperation compared with techniques relying exclusively on sutures, cartilage scoring, incisionless or suture + flap approaches to otoplasty. Nevertheless, whilst the optimal approach to otoplasty appears to be suture + cartilage-scoring and/or cartilage-scoring + flap hybrid approaches, this is not a conclusive statement due to the scarcity of high-quality RCTs, and therefore, definitive conclusions cannot yet be drawn. Robust, long-term RCTs directly comparing all major otoplasty modalities are essential to establish the most effective and durable approach.

## Funding

None.

## Declaration of competing interest

None.

## Appendix Supplementary materials

Image, application 1
