# Supplementary material for: Otoplasty for prominent ear: A systematic review of surgical techniques
Source: JPRAS Open. 2026 Feb 23;49:221–33. doi: 10.1016/j.jpra.2026.02.013 (PMC12996986; doi:10.1016/j.jpra.2026.02.013)
Supplement: Supplementary file 1 [file mmc1.docx]

**Otoplasty for Prominent Ear: A Systematic Review of Surgical Techniques**

Authors - Hamish Thomson (HT)^1^, Jack Gosden (JG)^1^, Anirban Mandal (AM)^2^

1 - School of Medicine, University of Liverpool, Liverpool, UK

2 - The Mersey Regional Burns and Plastic Surgery Unit, Mersey and West Lancashire Teaching Hospitals NHS Trust, Knowsley, UK

Author responsible for editorial correspondence:

Hamish Thomson

School of Medicine, University of Liverpool, Liverpool, UK

hamish.thomson25@icloud.com

07960419408

Word count (from introduction to end of conclusion) – 2,602

Number of figures - 3

Number of tables - 4

Number of references – 41

**Abstract**

Prominent ear is a common auricular malformation that can have long-term psychosocial consequences on affected individuals. Otoplasty remains the standard surgical corrective treatment; however, the optimal technique has long been debated, with more than 200 methods described in the literature. This systematic review, conducted in accordance with PRISMA guidelines, aimed to evaluate the available evidence on the most effective otoplasty approach. The outcomes assessed across different techniques (exclusive suture, exclusive cartilage-scoring, incisionless, suture + flap, suture + cartilage-scoring, and cartilage-scoring + flap hybrids) included: **a) number of patients suffering a complication, b)** recurrence, **c)** reoperation, **d)** infection, **e)** keloid or hypertrophic scar formation, and **f)** haematoma. A search of PubMed and MEDLINE identified 412 papers. Following PICOT framework-guided screening, application of inclusion and exclusion criteria, and quality assessment, 2**2** studies were included: 1**9** retrospective and **three** prospective cohort studies. Current medical literature suggests that suture + cartilage-scoring and cartilage-scoring + flap **hybrid** methods, may be associated with lower rates of recurrence, **and reoperation** when compared to single-technique **or suture + flap** approaches. However, **high**-quality, long-term **randomised control trials are** required to determine the superior technique for otoplasty.

**Keywords**

otoplasty; prominent ear; superior; technique; comparison

**Introduction**

Protruding ear is one of the most common auricular deformities affecting the Caucasian population, with approximately 5% believing that their ears excessively protrude (1). It features inadequate folding of the antihelix and/or increased conchal depth, leading to excessive projection of the auricle, typically >2cm from the mastoid, or with an auriculocephalic angle >25°. The aesthetic and psychosocial consequences of protruding ear can be detrimental to the individual and is known to lower self-esteem, increase social anxiety and in the case of children, increase their susceptibility to bullying (1).

Otoplasty is the mainstay of treatment to correct prominent ear. With over 200 otoplasty techniques being described in the current literature, it can be challenging for surgeons to select the **optimal** method for their patients (2). An otoplasty typically starts with a postauricular incision being made within the auriculomastoid sulcus to access the auricular cartilage. From this point, many techniques exist to recreate the antihelical fold and/or set back the conchal bowl (3).

- **Cartilage-sparing (suture-based) techniques** such as Mustardé (1963) (4) utilise mattress sutures to create the anti-helical fold. To achieve a balanced correction, this method is often performed in combination with another suture-based technique, Furnas (1968) (5), which employs concha-mastoid mattress sutures to set back the auricle by narrowing the mastoid helical angle. This **approach** is **best utilised** when the auricular cartilage is immature and pliable such as that found in children, however in older patients with firmer cartilage, recurrence is more common due to suture cut-through and/or cartilage memory (6).
- **Cartilage-scoring techniques**, pioneered by Stensröm (7), Crikelair (8) and Chongchet (9) exploit the **bio-mechanical** mechanism that cartilage **over time, naturally** bends **away from** the side of scoring. Scoring, or thinning the cartilage allows controlled formation of an anti-helical fold which is particularly advantageous in patients with thick or rigid cartilage. Excessive or uneven scoring risks the production of sharp edges and/or chondronecrosis (6).
- **Incisionless techniques** first described by Fritsch (1995) (10) revolve around the percutaneous placement of mattress sutures through needle punctures, negating the need for large skin incisions and theoretically reducing the risk of haematoma, necrosis and keloid/hypertrophic scarring. Similar to suture-based techniques, it is optimal to be used in the younger population (10).
- **Suture-postauricular fascial flap hybrid** is a method piloted by Horlock et al. (2001) (11) that introduces a postauricular flap to provide a vascularised layer over the sutures, thereby theoretically reducing the risk of extrusion and recurrence, a notorious limitation of using sutures exclusively (11).
- **Suture-cartilage scoring hybrids** combine posterior suturing with minimal anterior cartilage-scoring to achieve a superior fold, while reducing the risks of suture extrusion associated with sutures alone, and the sharp edges or weakening linked to cartilage-only method (12).
- **Cartilage scoring-flap hybrids** as described by Scuderi et al. (2006) (13) combine posterior chondro-muscular flap advancement with anterior cartilage **scoring**. The flap narrows the auriculocephalic angle, and adjusts the auricular slope, while cartilage scoring or cutting recreates the antihelical fold. This dual approach enhances stability and contour while reducing risks of recurrence, or unnatural rigidity that can be seen with single-technique methods (13).

Several complications are associated with otoplasty, both in the short term such as bleeding, infection, haematoma and necrosis, and the long term, including **recurrence, reoperation**, suture extrusion, asymmetry and **poor aesthetic** results. These complications increase the risk of prolongating the negative psychological impact that prominent ear can have on patients. There is no up-to-date comparison between techniques, necessitating the need for a review (2).

This systematic review was conducted in accordance with PRISMA guidelines (14), and followed the population, **intervention, comparison, outcome** and time (PICOT) framework (15). The review compares traditional techniques, exclusive suture or cartilage-scoring methods, with more recent approaches, including incisionless, and hybrid techniques (suture + flap, suture + cartilage-scoring, and cartilage-scoring + flap). The study population comprised patients undergoing otoplasty for the correction of prominent ear. The primary intervention of interest was the use of suture-only or cartilage-scoring techniques, with comparators including incisionless and hybrid approaches. **Outcomes** assessed **included a) number of patients suffering a complication, b)** recurrence, **c**) reoperation, **d**) infection, **e**) keloid or hypertrophic scar formation, and **f**) haematoma. **These outcomes** were evaluated in both the short term (<1 year) and long term (>1 year). This review aims to provide a reference for plastic surgeons in selecting the most appropriate otoplasty technique for patients with prominent ear. We hypothesised that suture-based techniques combined with a postauricular fascial flap represent the most effective method for achieving durable correction.

**Methodology**

A systematic search of the medical databases PubMed and MEDLINE (16, 17) was conducted without the assistance of a medical librarian on 1 September 2025, using search strategies specific to each database, adhering to PRISMA guidelines (14) and following the PICOT framework (15). PubMed and MEDLINE were selected for their extensive coverage of high-quality studies published in the fields of medicine and surgery. The MEDLINE search was performed using the OVID search engine (18). Only papers published between 1 January 2001, and 1 September 2025 were selected to ensure the review aligns with **modern** otoplasty techniques. The precise search terms and Boolean operators used for each database are detailed in the appendix, within Tables 1 and 2. All types of study were included in the search strategy. Following the search, all identified studies were uploaded to the latest version of the AI systematic review software Rayyan (19) and 2 authors (H.T. and A.M.) evaluated the abstracts against the PICOT framework-guided (15) inclusion criteria. Additionally, the bibliography of each article was screened to identify further relevant studies.

The selection process was guided by the PICOT framework to ensure optimal comparison between otoplasty outcomes (15):

- **Population (P) –** Patients undergoing otoplasty to correct prominent ear.
- **Intervention (I) –** Otoplasty exclusively using cartilage-sparing (suture-based) or cartilage-scoring techniques.
- **Comparison (C) –** Otoplasty using incisionless, or suture-flap, suture-cartilage scoring or cartilage scoring-flap hybrids.
- **Outcome (O) – a) Number of patients suffering a complication, b)** Recurrence, **c**) Reoperation, **d**) Infection, **e**) Keloid or hypertrophic scar, **f**) Haematoma
- **Time (T) –** Both short-term (≤ 1 year) and long term (≥ 1 year)

**Inclusion Criteria**

The paper must:

- Include primary data.
- Include human participants.
- Be comprised of ≥ 50 patients.
- Have an identifiable study design.
- Involve one of, **or** a combination of suture-based, cartilage-scoring, incisionless or hybrid technique to perform otoplasty to correct prominent ear.
- Be available in English.
- Be fully accessible to the authors.
- Be a full study.
- Meet high ethical standards.

**Exclusion Criteria**

The paper must not:

- Be a systematic or literature review.
- Be published **prior** to 1 January 2000.
- Have ≤ 50 patients*.

Summarised in Figure 1 (20), the original search identified 412 studies (PubMed = 165; MEDLINE = 247). Following the removal of 162 duplicates, 250 papers were independently assessed by authors H.T and A.M. against the inclusion and exclusion criteria, and PICOT framework. 20**9** papers were **excluded, and** one additional duplicate was identified, leaving 4**0** papers to be put through quality assessment. The quality and risk of bias in each study were evaluated using the Risk of Bias in Non-Randomised Studies of Interventions, Version 2 (ROBINS-I V2) tool (21). Studies with a moderate risk of bias were accepted due to the limited availability of randomised controlled trials (RCTs**). A total of 19 papers were excluded during the quality assessment process, leaving 21 papers included in the final review**.

The characteristics of each study are detailed in Table 3. Patients that underwent each method of otoplasty were identified and compiled into a sub-analysis. Patient characteristics, confounding factors, and each of the primary outcomes; **a**) **Number of patients suffering a complication**, **b**) recurrence, **c**) reoperation, **d**) infection, **e**) keloid **or hypertrophic scar** and **f**) haematoma were stratified for each method of otoplasty and displayed in Table 4. Studies reporting mean values were grouped together and a cumulative mean was calculated. Similarly, studies reporting median values were grouped and a cumulative median was calculated.

**Results**

**Study Characteristics**

A total of 2**1** studies were included in this review, comprising 1**8** retrospective cohort studies (12, 13, 22-37), and **three** prospective cohort studies (38-40). The studies were conducted across a diverse range of countries. Sample sizes ranged from **50** to 705 (12, 22) with a median of 10**4** and a mean of **136.2** participants. Only patients who underwent an otoplasty technique relevant to this review were included in the reported sample sizes. The number of ears analysed ranged from **90** to 1380, with a median of **214** and a mean of **254.5**. All studies were single-centre in design. Among studies reporting follow-up duration using median values, the pooled median was 12.65 months (range 9–36), while the pooled mean follow-up duration was **17 +/- 8.5** months.

**Method Of Otoplasty**

A total of 812 patients underwent otoplasty using exclusively suture-based techniques, of whom 381 (46.9%) were male. Cartilage-scoring techniques alone were performed in **590** patients, including **283** **(48%) males**. The incisionless approach was used in 60 patients, with 34 (56.6%) males. A suture + flap hybrid technique was employed in 263 patients, including 201 (76.4%) males. Suture + cartilage-scoring hybrids were used in 1,074 patients, with 406 (37.8%) males. Finally, a cartilage-scoring + flap hybrid was performed in 55 patients, 38 (47.5%) of whom were male.

**Number of Patients Suffering a Complication**

**The highest proportion of patients suffering a complication was associated with the exclusive use of suture-based techniques, with 25.6% of patients affected; incisionless techniques followed with a rate of 25%. Exclusive cartilage-scoring approaches resulted in complications in 20.5% of patients. In contrast, hybrid techniques demonstrated lower numbers of patients suffering a complication: 13.7% for suture + flap hybrids, 10.4% for suture + cartilage-scoring hybrids, and 3.6% for cartilage-scoring + flap hybrids. The total number of patients suffering a complication from each method of otoplasty is summarised in Figure 2.**

**Recurrence**

Recurrence was consistently reported across the included studies. Overall, recurrence was ≤ 10% for all otoplasty techniques, with the incisionless method reporting the highest (n=10%). Among patients who underwent isolated suture or cartilage-scoring techniques, recurrence was observed in 5.5% and **8.3%** of cases, respectively. The suture + flap hybrid technique was associated with a recurrence rate of 9.1%. The lowest recurrence rates were reported with suture + cartilage-scoring (3.4%) and cartilage-scoring + flap (3.6%) hybrid techniques. The recurrence rates for each method of otoplasty are **displayed** in Figure 2.

**Reoperation**

Reoperation rates, whether due to recurrence, asymmetry, patient dissatisfaction, or other concerns, were consistently reported across the included studies. The highest rate was observed in the **incisionless** cohort, with **10%** of patients requiring a secondary procedure. **E**xclusive suture and cartilage-scoring techniques required further surgery in 4.6% and **6.6%** of cases, respectively; suture + flap hybrids and suture + cartilage-scoring hybrids in 7.9% and 3.9%, respectively. The lowest rate was reported in the cartilage-scoring + flap hybrid group, with 3.6% of patients requiring reoperation. The reoperations rates for each method of otoplasty are **shown** in Figure **3**.

**Infection**

Overall, infection rates were low across all methods. No postoperative infections were reported among the hybrid techniques (suture + flap, suture + cartilage-scoring, and cartilage-scoring + flap), or incisionless methods. Exclusive **cartilage-scoring and suture-based** techniques each reported an infection rate of **1.02% and** 0.9% **respectively**.

**Keloid Or Hypertrophic Scar Formation**

Similar to infections, **keloid or hypertrophic scar formation was** relatively uncommon across the review. No cases were reported in the incisionless cohort or among patients undergoing the suture + flap or cartilage-scoring + flap hybrid techniques. Exclusive cartilage-scoring resulted in keloid or hypertrophic scars in **1.7%** of patients. Exclusive suture-based techniques and suture + cartilage-scoring hybrids demonstrated comparable rates, at 2.2% and 2.1%, respectively.

**Haematoma**

**H**aematomas were **also** uncommon. No cases were observed in the cartilage-scoring + flap hybrid group nor those undergoing an incisionless technique. Haematomas occurred in 0.6% of patients treated with exclusive suture techniques and **0.5**% with exclusive cartilage-scoring. The suture + flap and suture + cartilage-scoring hybrids reported rates of 0.4% and 0.1%, respectively.

**Discussion**

This systematic review aimed to explore the available medical literature on the optimal technique of otoplasty for correction of prominent ear. Several key observations were made in the review.

The literature indicates that **suture + cartilage-scoring and cartilage-scoring + flap** hybrid techniques represent the most effective methods of otoplasty. These techniques were associated with **lower rates of recurrence and reoperation** compared to other methods. Notably, the suture + cartilage-scoring approach had the largest reported sample size (n = 1,074), strengthening the evidence for its efficacy in the management of prominent ear. **Although fewer patients undergoing suture–flap hybrid otoplasty experienced complications compared with exclusively suture-based and cartilage-scoring techniques, a higher proportion of these complications being recurrence and/or reoperation was observed. This suggests that the suture-flap hybrid technique may be associated with the greatest risk of recurrence and/or reoperation amongst hybrid methods of otoplasty, which differs** from our hypothesis that the suture + flap hybrid would have superior outcomes. The evidence base for incisionless **otoplasty** remains limited due to small sample size and reported outcomes **appearing** less favourable compared with **other** methods. **This is emphasised by the** incisionless technique **demonstrating** the highest rates of recurrence and reoperation.

Traditional techniques, such as the exclusive use of sutures and/or cartilage-scoring, demonstrated **higher incidences of** recurrence and reoperation **than suture + cartilage-scoring and cartilage-scoring + flap hybrid techniques, however, demonstrated a lower recurrence and reoperation rate than incisionless and suture + flap hybrid methods.** Notably, the exclusive use of sutures was associated with the **highest proportion of patients experiencing a complication in the review**. **This is likely due to** the increased incidence of minor complications such as suture extrusion and/or irregularity of the contour fold. This suggests that, while traditional techniques may provide adequate correction of prominent ear, combining them either with each other, or **cartilage-scoring** with a vascularised flap, appears to **reduce the number of patients suffering a complication,** and thereby improve overall patient outcomes.

In comparison to a review on the same topic, Sadhra et al. (2017) (41) evaluated the average incidence of complications following otoplasty and identified the exclusive use of sutures as the method associated with the highest rates of recurrence and reoperation (41). Unlike the present review, their analysis was limited to suture-only, cartilage-scoring, and suture + cartilage-scoring hybrid techniques. Moreover, Sadhra et al. predates the publication of several studies included in the current review. This emphasises the need for an updated review that incorporates the full range of otoplasty methods currently in practice.

To accurately determine the superior method of otoplasty **or confirm suture + cartilage-scoring and/or cartilage-scoring + flap methods as the superior technique**, long-term RCTs are required, with comprehensive reporting on patient characteristics, and clinical outcomes per patient. All studies included in this review adhered to the ethical guidelines, provided appropriate follow-up care and, in several cases, reported long-term outcome monitoring, reflecting a commitment to the highest principles of medical research ethics.

**Conclusion**

In conclusion, although this review **is** limited by the absence of randomised controlled trials (RCTs), inconsistent stratification and outcome reporting, and unequal sample sizes within the comparative groups in the sub-analysis, the available evidence suggests that **suture + cartilage-scoring and/or cartilage-scoring + flap hybrid approaches** to otoplasty may offer superior outcomes. These methods are generally associated with lower rates of **recurrence, and reoperation** compared with techniques relying exclusively on sutures, cartilage scoring, **incisionless or suture + flap approaches to otoplasty**. Nevertheless, **whilst** the optimal **approach to otoplasty appears to be suture + cartilage-scoring and/or cartilage-scoring + flap hybrid approaches, this is not a conclusive statement** due to the scarcity of high-quality RCTs, and definitive conclusions cannot yet be drawn. Robust, long-term RCTs directly comparing all major otoplasty modalities are essential to establish the most effective and durable approach.

**Acknowledgements**

None.

**Funding**

None.

**Conflict Of Interest Statement**

None.

**Ethical Approval**

Not required.

**Additional Information**

This review was not registered. A protocol was not prepared.

**References**

1. Jones ES, Gibson JAG, Dobbs TD, Whitaker IS. The psychological, social and educational impact of prominent ears: A systematic review. Journal of Plastic, Reconstructive & Aesthetic Surgery. 2020;73(12):2111-20. 2/9/2025. <https://www.sciencedirect.com/science/article/pii/S1748681520302588>.

2. Limandjaja GC, Breugem CC, Mink van der Molen AB, Kon M. Complications of otoplasty: a literature review. J Plast Reconstr Aesthet Surg. 2009;62(1):19-27. 2/9/2025. <https://pubmed.ncbi.nlm.nih.gov/18952516/>.

3. British Association of Plastic RaAS. Prominent Ears 2025 [2/9/2025]. Available from: <https://www.bapras.org.uk/public/patient-information/surgery-guides/ear-surgery/prominent-ears>.

4. Mustarde JC. The correction of prominent ears using simple mattress sutures. Br J Plast Surg. 1963;16:170-8. 2/9/2025. <https://pubmed.ncbi.nlm.nih.gov/13936895/>.

5. Furnas DW. Correction of prominent ears by conchamastoid sutures. Plastic Reconstructive Surgery. 1968;42(3):189-93. 2/9/2025. <https://pubmed.ncbi.nlm.nih.gov/4878456/>.

6. Naumann A. Otoplasty - techniques, characteristics and risks. GMS Curr Top Otorhinolaryngol Head Neck Surg. 2007;6:Doc04. 2/9/2025. <https://pmc.ncbi.nlm.nih.gov/articles/PMC3199845/>.

7. Stenstroem SJ. A "Natural” Technqiue For Correction Of Congenitally Prominent Ears. Plastic Reconstructive Surgery. 1963;32:509-18. 6/9/2025. <https://pubmed.ncbi.nlm.nih.gov/14078273/>.

8. Crikelair GF, Cosman B. Another Solution For The Problem Of The Prominent Ear. Annals of Surgery. 1964;160(2):314-24. 2/9/2025. <https://pmc.ncbi.nlm.nih.gov/articles/PMC1408820/>.

9. Chongchet V. A Method Of Antihelix Reconstruction. British Journal of Plastic, Reconstructive and Aesthetic Surgery. 1963;16:268-72. 2/9/2025. <https://pubmed.ncbi.nlm.nih.gov/14042756/>.

10. Fritsch MH. Incisionless otoplasty. Laryngoscope. 1995;105(5 Pt 3 Suppl 70):1-11. 2/9/2025. <https://pubmed.ncbi.nlm.nih.gov/7760682/>.

11. Horlock N, Misra A, Gault DT. The postauricular fascial flap as an adjunct to Mustardé and Furnas type otoplasty. Plast Reconstr Surg. 2001;108(6):1487-90; discussion 91. 2/9/2025. <https://pubmed.ncbi.nlm.nih.gov/11711914/>.

12. Binet A, El Ezzi O, De Buys Roessingh A. A retrospective analysis of complications and surgical outcome of 1380 ears: Experience review of paediatric otoplasty. Int J Pediatr Otorhinolaryngol. 2020;138:110302. 6/9/2025.

13. Scuderi N, Tenna S, Bitonti A, Vonella M. Repositioning of posterior auricular muscle combined with conventional otoplasty: a personal technique. J Plast Reconstr Aesthet Surg. 2007;60(2):201-4. 6/9/2025.

14. Page MJ, McKenzie JE, Bossuyt PM, Boutron I, Hoffmann TC, Mulrow CD, et al. The PRISMA 2020 statement: an updated guideline for reporting systematic reviews. BMJ (Online). 2021;372:n71-n. 1/9/2025. <https://www.bmj.com/content/372/bmj.n71>.

15. Riva J, Malik K, Burnie S, Endicott A, Busse J. What is your research question? An introduction to the PICOT format for clinicians. Journal of the Canadian Chiropractic Association. 2012;56(3):167-71. 4/4/2025.

16. PubMed - National Library of Medicine [Internet]. 2025 [cited 1/9/2025]. Available from: <https://pubmed.ncbi.nlm.nih.gov>.

17. MEDLINE: Medical Literature Analysis and Retrieval System Online [Internet]. 2025 [cited 1/9/2025]. Available from: <https://www.nlm.nih.gov/medline/>.

18. Ovid: Your Research Platform [Internet]. 2025 [cited 1/9/2025]. Available from: <https://ovidsp.ovid.com/>.

19. Ouzzani M HH, Fedorowicz Z, Elmagarmid A. Rayyan – a web and mobile app for systematic reviews: Qatar Computing Research Institute (QCRI); 2016 [Available from: <https://rayyan.ai>.

20. PRISMA. PRISMA 2020 flow diagram template. 2020.

21. Tools RoB. ROBINS-I V2 tool: Riskofbias.info; 2016 [10/11/24]. Available from: <https://drive.google.com/file/d/1LCc9_KFIpdP3_uR56M-7ngO0zppxpAMS/view>.

22. Grella R, Molle M, Crisci E, Grella E, Nicoletti MM, Pieretti G. Modified Anterior Scoring Otoplasty Without Sutures: Description and Case Series. Aesthetic Plast Surg. 2025;49(11):2949-55. 6/9/2025.

23. Gualdi A, Cambiaso-Daniel J, Negrini FC, Giordano S. Double Triangular Cartilage Excision in Otoplasty Revisited. Facial Plast Surg. 2025;41(2):159-65. 6/9/2025.

24. Gilron S, Weiss N, Kouniavski E, Egozi D, Dolgunin L, Benkler M. Cartilage-sparing otoplasty - 288 ears in 3 years. J Plast Reconstr Aesthet Surg. 2024;99:209-20. 6/9/2025.

25. Kadhum M, Atherton S, Jawad A, Wilson-Jones N, Javed MU. A Retrospective Analysis of Pinnaplasty Outcomes: The Welsh Experience. Facial Plast Surg. 2024;40(4):499-504. 6/9/2025.

26. García-Purriños F, Raposo A, Guilllén A, Calero J, Giribet A, Barrios A. Otoplasty Using the Combined Mustardé-Furnas Technique: Satisfaction and Objective Results. Aesthet Surg J. 2019;39(10):Np411-np5. 6/9/2025.

27. Brian T, Cheng PT, Loo SS. Audit of 117 otoplasties for prominent ear by one surgeon using a cartilage-cutting procedure. ANZ J Surg. 2019;89(3):E66-e70. 6/9/2025.

28. Ersen B. A Modification of the Posterior Perichondrio-Adipo-Dermal Flap for Protruding Ear Correction: A Customized Technique. Ann Plast Surg. 2019;83(5):500-6. 6/9/2025.

29. Park C, Jeong TW. Antihelical shaping of prominent ears using conchal cartilage-grafting adhesion. Laryngoscope. 2012;122(6):1238-45. 6/9/2025.

30. Maricevich P, Gontijo de Amorim NF, Duprat R, Freitas F, Pitanguy I. Island technique for prominent ears: an update of the Ivo Pitanguy clinic experience. Aesthet Surg J. 2011;31(6):623-33. 6/9/2025.

31. Schaverien MV, Al-Busaidi S, Stewart KJ. Long-term results of posterior suturing with postauricular fascial flap otoplasty. J Plast Reconstr Aesthet Surg. 2010;63(9):1447-51. 6/9/2025.

32. Olivier B, Mohammad H, Christian A, Akram R. Retrospective study of the long-term results of otoplasty using a modified Mustardé (cartilage-sparing) technique. J Otolaryngol Head Neck Surg. 2009;38(3):340-7. 6/9/2025.

33. Scharer SA, Farrior EH, Farrior RT. Retrospective analysis of the Farrior technique for otoplasty. Arch Facial Plast Surg. 2007;9(3):167-73. 6/9/2025.

34. Salgarello M, Gasperoni C, Montagnese A, Farallo E. Otoplasty for prominent ears: a versatile combined technique to master the shape of the ear. Otolaryngol Head Neck Surg. 2007;137(2):224-7. 6/9/2025.

35. Mandal A, Bahia H, Ahmad T, Stewart KJ. Comparison of cartilage scoring and cartilage sparing otoplasty--A study of 203 cases. J Plast Reconstr Aesthet Surg. 2006;59(11):1170-6. 6/9/2025.

36. Bulstrode NW, Huang S, Martin DL. Otoplasty by percutaneous anterior scoring. Another twist to the story: a long-term study of 114 patients. Br J Plast Surg. 2003;56(2):145-9. 6/9/2025.

37. Yugueros P, Friedland JA. Otoplasty: the experience of 100 consecutive patients. Plast Reconstr Surg. 2001;108(4):1045-51; discussion 52-3. 6/9/2025.

38. Uyar I, Aksam E, Kopal C, Kapı E. Comparison of Transcutaneous Fixation-Assisted Method with Classical Needle-Assisted Method in Prominent Ear Surgery. Aesthetic Plast Surg. 2023;47(1):189-98. 6/9/2025.

39. Haytoglu S, Haytoglu TG, Bayar Muluk N, Kuran G, Arikan OK. Comparison of two incisionless otoplasty techniques for prominent ears in children. Int J Pediatr Otorhinolaryngol. 2015;79(4):504-10. 6/9/2025.

40. Toplu Y, Sapmaz E, Firat C, Toplu SA. Clinical results and health-related quality of life in otoplasty patients using cartilage resection and suturing methods. Eur Arch Otorhinolaryngol. 2014;271(12):3147-53. 6/9/2025.

41. Sadhra SS, Motahariasl S, Hardwicke JT. Complications after prominent ear correction: A systematic review of the literature. J Plast Reconstr Aesthet Surg. 2017;70(8):1083-90.

**Appendix**

**Table 1 – PubMed Search Strategy**

Date – 01/09/2025

| **Number** | **Term** | **Results** |
| --- | --- | --- |
| **#1** | (otoplasty [tiab]) | 674 |
| **#2** | (prominent ears [tiab]) OR (prominent ear [tiab]) | 622 |
| **#3** | #1 OR #2 | 1,042 |
| **#4** | (management [tiab]) | 1,737,902 |
| **#5** | (comparison [tiab]) | 1,325,810 |
| **#6** | (outcome [tiab]) | 1,440,532 |
| **#7** | #4 OR #5 OR #6 | 4,195,520 |
| **#8** | #3 AND #7 | 191 |
| **#9** | #3 AND #7 Filter: from 1/1/2001 – 1/9/2025 | 165 |

**Table 2 – MEDLINE Search Strategy**

Date – 01/09/2025

| **Number** | **Term** | **Results** |
| --- | --- | --- |
| **#1** | otoplasty.mp. | 674 |
| **#2** | prominent ears.mp. OR prominent ear.mp. | 621 |
| **#3** | #1 OR #2 | 1,041 |
| **#4** | management.mp. | 1,898,364 |
| **#5** | comparison.mp. | 1,349,856 |
| **#6** | outcome.mp. | 2,581,434 |
| **#7** | #4 OR #5 OR #6 | 5,327,788 |
| **#8** | #3 AND #7 | 287 |
| **#9** | Limit #8 to yr=”2001-2025” | 247 |

**Figure Legends**

**Figure 1 –** PRISMA flowchart.

**Figure 2 – Number of patients suffering a complication.**

**Figure 3 –** Recurrence **and reoperation** for each method of otoplasty.

**Tables**

**Table 3 – Study Characteristics**

| Study | Location | Single or Multi Centre | Study Type | Sample | Ears | Median (Range) / Mean (+/-SD) Age, years | Gender, n (%) | Method(s) of Otoplasty, n (%) | Follow-Up, Median (Range) or Mean (+/- SD), months |
| --- | --- | --- | --- | --- | --- | --- | --- | --- | --- |
| (Grella, 2025) | Italy | Single | Retrospective | 50 | 100 | 20 +/- 11.5 | M – 24 (48)  F – 26 (52) | CS – 50 (100) | 33.9 +/- 16.1 |
| (Gualdi, 2025) | Italy | Single | Retrospective | 146 | 292 | 23.2 +/- 5.3 | M – 81 (55.5)  F – 65 (44.5) | CS – 146 (100) | 22 |
| (Gilron, 2024) | Israel | Single | Retrospective | 147 | 288 | 16.6 +/- 9.9 | M – 56 (38.1)  F – 91 (61.9) | SB – 147 (100) | 24.3 +/- 11.2 |
| (Kadhum, 2024) | UK | Single | Retrospective | 203 | 372 | 13 +/- 4 | M – 103 (51)  F – 100 (49) | SB – 194 (96)  CS – 9 (4) | 12 |
| (Uyar, 2023) | Turkey | Single | Prospective | 52 | 104 | 14.3 +/- 5.8 | M – 20 (38.5)  F – 32 (61.5) | SB – 52 (100) | 12 |
| (Binet, 2020) | Switzerland | Single | Retrospective | 705 | 1380 | 10.4 +/- 2.9 | M – 266 (37.8)  F – 439 (62.2) | S+CS – 705 (100) | 132 +/- 72 |
| (García-Purriños, 2019) | Spain | Single | Retrospective | 172 | 343 | 16.2 +/- 14 | M – 61 (35.3)  F – 111 (64.5) | SB – 172 (100) | 18 |
| (Brian, 2019) | NZ | Single | Retrospective | 64 | 117 | 9.5 +/- 4.2 | M – 29 (45.3)  F – 35 (54.7) | CS – 64 (100) | 32.7 +/- 17.1 |
| (Ersen, 2019) | Turkey | Single | Retrospective | 162 | 322 | 28.3 (18-40) | M – 141 (87)  F – 21 (13) | S+PF – 162 (100) | 22.5 +/- 7.5 |
| (Haytoglu, 2015) | Turkey | Single | Prospective | 60 | 112 | 8.3 +/- 2.8 | M – 34 (56.6)  F – 26 (43.3) | INCL – 60 (100) | NR |
| (Toplu, 2014) | Turkey | Single | Prospective | 77 | 132 | 14.6 +/- 6.4 | M – 18 (45)  F – 22 (55) | SB – 40 (51.9)  CS – 37 (48.1) | 8 |
| (Park, 2012) | SK | Single | Retrospective | 66 | 90 | 23.7 +/- 10.1 | M – 27 (40.9)  F – 39 (59.1) | S+CS – 66 (100) | 62.4 +/- 12 |
| (Marichevic, 2011) | Brazil | Single | Retrospective | 111 | 218 | 28.2 +/- 15 | M – 31 (28)  F – 80 (72) | CS – 111 (100) | NR |
| (Schaverian, 2010) | UK | Single | Retrospective | 60 | 112 | 8.5 +/- 2.5 | M – 34 (56.6)  F – 26 (43.3) | S+PF – 60 (100) | 46.8 +/- 18.9 |
| (Olivier, 2009) | Canada | Single | Retrospective | 104 | 203 | 7.1 +/- 3.5 | M – 64 (62)  F – 40 (38) | SB – 104 (100) | 99 |
| (Scharer, 2007) | USA | Single | Retrospective | 75 | 144 | 23.9 +/- 15.8 | M – 40 (53)  F – 35 (47) | S+CS – 73 (97.3)  N/A – 2 (2.7) | 12 +/- 21.4 |
| (Salgarello, 2007) | Italy | Single | Retrospective | 135 | 266 | 13 +/- 7.5 | M – 51 (37.7)  F – 84 (62.3) | S+CS – 135 (100) | 60 +/- 33 |
| (Scuderi, 2007) | Italy | Single | Retrospective | 55 | 103 | 14 (6-55) | M – 38 (47.5)  F – 42 (52.5) | CS+F – 55 (100) | 14.3 (12-36) |
| (Mandal, 2006) | UK | Single | Retrospective | 203 | 406 | 9 (5-16) | M – 127 (62.6)  F – 76 (37.4) | SB – 94 (46.3)  CS – 68 (33.5)  S+PF – 41 (20.2) | 11 (9-36) |
| (Bulstrode, 2003) | UK | Single | Retrospective | 114 | 214 | 18.3 +/- 15.8 | M – 57 (50)  F – 57 (50) | CS – 114 (100) | 47 +/- 26.3 |
| (Yugueros, 2001) | USA | Single | Retrospective | 100 | 193 | 38 +/- 15.5 | M – 29 (29)  F – 71 (71) | S+CS -100 (100) | 40 +/- 51 |

Key – CS –Cartilage Scoring Technique; CS+F – Cartilage Scoring + Flap Hybrid; F – Female; M – Male; INCL – Incisionless Technique; N/A – Unknown; NR – Not Reported; NZ – New Zealand; SB – Suture-Based Technique; SK – South Korea; ; S+CS – Suture + Cartilage Scoring Hybrid; S+PF – Suture-Postauricular Flap Hybrid; UK – United Kingdom; USA – United States of America

**Table 4 – Sub Analysis of Otoplasty Techniques**

|  | Suture-based (n=812) | Cartilage-scoring  (n=590) | Incisionless  (n=60) | Suture + Flap  (n=263) | Suture + Cartilage-scoring  (n=1074) | Cartilage-scoring + Flap  (n=55) |
| --- | --- | --- | --- | --- | --- | --- |
| Age  Cumulative Median (Range)  Cumulative Mean (+/- SD) | 11 (9-36)  13.6 +/- 7.3 | 9 (5-16)  18.9 +/- 9.7 | -  8.3 +/- 2.8 | 18.7 (5-40)  8.5 +/- 2.5 | -  21.8 +/- 13.1 | 14 (6-55)  - |
| Gender, n (%)  Male  Female | 381 (46.9)  431 (53.1) | 283 (48)  307 (52) | 34 (56.6)  26 (43.3) | 201 (76.4)  62 (23.6) | 406 (37.8)  668 (62.2) | 38 (47.5)  42 (52.5) |
| Ears, n | 1566 | 1141 | 112 | 516 | 2072 | 103 |
| Follow up, months  Cumulative Median (Range)  Cumulative Mean (+/- SD) | 11 (9-36)  28.9 +/- 11.2 | 11 (9-36)  28.3 +/- 11.9 | -  6 | 11 (9-36)  34.7 +/- 13.2 | -  50.5 +/- 37.9 | 14.3 (12-36)  - |
| Confounding Factors, n (%)  Smoker | 20 (2.5) | 8 (1.4) | NR | NR | NR | NR |
| Patients suffering a  complication, n (%) | 208 (25.6) | 121 (20.5) | 15 (25) | 36 (13.7) | 112 (10.4) | 2 (3.6) |
| Recurrence, n (%) | 45 (5.5) | 49 (8.3) | 6 (10) | 24 (9.1) | 36 (3.4) | 2 (3.6) |
| Reoperation, n (%) | 37 (4.6) | 39 (6.6) | 6 (10) | 21 (7.9) | 42 (3.9) | 2 (3.6) |
| Infection, n (%) | 8 (0.9) | 6 (1.02) | 0 | 0 | 0 | 0 |
| Keloid or hypertrophic scar, n (%) | 18 (2.2) | 10 (1.7) | 0 | 0 | 22 (2.1) | 0 |
| Haematoma, n (%) | 9 (1.1) | 3 (0.5) | 0 | 1 (0.4) | 1 (0.1) | 0 |

Key –NR – Not reported
